# Echocardiographic characteristics of pulmonary artery involvement in Takayasu arteritis

**DOI:** 10.1111/echo.13464

**Published:** 2017-01-31

**Authors:** Wei Jiang, Yuanhua Yang, Xiuzhang Lv, Yidan Li, Zhanhong Ma, Jifeng Li

**Affiliations:** ^1^ Department of EchocardiographyHeart CenterCapital Medical UniversityChaoyang HospitalBeijingChina; ^2^ Department of Respiratory and Critical Care MedicineCapital Medical UniversityChaoyang HospitalBeijingChina; ^3^ Department of RadiologyCapital Medical UniversityChaoyang HospitalBeijingChina

**Keywords:** computed tomographic pulmonary angiography, echocardiography, pulmonary artery, Takayasu arteritis

## Abstract

**Background:**

Up to 50% patients with Takayasu arteritis have pulmonary artery involvement. Hence, the early identification of pulmonary artery involvement to facilitate prompt treatment is required.

**Methods:**

This retrospective study was performed in patients diagnosed with Takayasu arteritis between January 2009 and January 2016. Pulmonary artery involvement was confirmed with computed tomographic pulmonary angiography. Images from transthoracic echocardiography in three windows (suprasternal right pulmonary artery long‐axis view, parasternal aortic short‐axis view, and subxiphoid view) were documented and analyzed.

**Results:**

A total of 27 patients had Takayasu arteritis and pulmonary artery involvement. Characteristic changes identified by echocardiography included luminal medium‐to‐high echogenic signals, stenosis, and occlusion, as well as intimal thickening. Left pulmonary artery involvement was revealed in the parasternal aortic short‐axis view. Right pulmonary artery involvement was best observed in the suprasternal right pulmonary artery long‐axis view, with complementary views from the parasternal aortic short‐axis and subxiphoid angles. Pulmonary trunk involvement was not observed in all three windows.

**Conclusions:**

Transthoracic echocardiography could be a useful noninvasive test to detect pulmonary artery involvement in patients with Takayasu arteritis.

## 1 Introduction

Takayasu arteritis is a chronic systemic vasculitis of unknown etiology.^1^ Affected patients can have stenosis, occlusion, or dilatation of the blood vessels, which can lead to substantial morbidities including stroke, aortic aneurysm, valvular heart disease and organ failure.^2^ Furthermore, it more likely involves the aorta and its primary branches, and up to 50% of patients can have pulmonary artery involvement.^3^, ^4^ However, due to unspecific clinical presentations, the diagnosis of pulmonary artery involvement is often delayed until patients develop symptoms of pulmonary hypertension, thrombosis, or right heart failure.^5^, ^6^, ^7^, ^8^ Thus, an effective method to evaluate pulmonary artery involvement is required.

The diagnosis of vascular involvement in Takayasu arteritis requires vascular imaging studies.^9^ Currently, arteriography, computed tomography, magnetic resonance imaging, and positron emission tomography are commonly used. However, these tests are either invasive or involve iodinated contrast infusion and radiation exposure. Previous studies have shown that transthoracic ultrasound could provide detailed vascular resolutions and mural involvements, which was useful in the evaluation of Takayasu arteritis.^10^, ^11^, ^12^, ^13^ However, these studies only reported the evaluations for carotid, brachiocephalic, and subclavian vascular changes. Furthermore, no previous study has investigated the role of ultrasound in the diagnosis of pulmonary artery involvement in Takayasu arteritis.

In this study, we investigated the features of transthoracic echocardiography on pulmonary artery involvement in patients with Takayasu arteritis. We have reported our results and discussed the clinical applications in this study.

## 2 Materials and Methods

### 2.1 Study design and patient selection

This study is a retrospective study performed in an academic teaching hospital. Study protocol was reviewed and approved by the hospital ethics committee. All patients were treated at our hospital from January 2009 to January 2016.

Patient selection criteria were as follows: (1) diagnosis of Takayasu arteritis based on the American College of Rheumatology Criteria^14^ and (2) diagnosis of pulmonary artery involvement based on computed tomographic pulmonary angiography (CTPA). Exclusion criteria were as follows: (1) pulmonary involvement only limited to lobules, subsegmental artery, but not the right and left pulmonary artery and (2) incomplete echocardiography records on pulmonary arteries.

### 2.2 Echocardiography

Transthoracic echocardiography was performed routinely as clinical diagnostic tests for patients with Takayasu arteritis by sonographers in our hospital. All sonographers received standard trainings and had the certificate to perform echocardiography. The equipment used was Philips iE33 ultrasound with a S5‐1 transducer (Philips, Amsterdam, The Netherlands) and GE Vivid I ultrasound with a 1.7‐3.4 MHz Transducer (General Electric, Fairfield, CT, USA). The main pulmonary artery and its main branches were evaluated in three windows: parasternal aortic short‐axis view, subxiphoid view, and suprasternal right pulmonary artery long‐axis view.^15^ Artery inner diameter and intima‐media thickness were documented. Pulmonary artery intimal thickening was identified when low‐moderate or high‐moderate echogenic areas were observed parallel and internal to the hyperechogenic vascular wall images. Color Doppler flow imaging was used to evaluate blood filling status in the pulmonary artery.

### 2.3 Statistical analysis

Numeric data were presented as mean±standard distribution. Categorical data were presented as percentage.

## 3 Results

A total of 27 patients were identified. All of them were diagnosed with Takayasu arteritis with pulmonary artery involvement. Their baseline characteristics including pulmonary and systemic artery involvements are listed in Table 1. Pulmonary artery involvement includes luminal stenosis, occlusions, tortuosity, dilation, or filling defects, and these were obtained through CTPA examinations.

**Table 1:** Baseline characteristics of patients with Takayasu arteritis

Variable	Numbers
Age, y, mean±SD	41.7±11.5
Women/men	21/6
Body mass index, kg/m, mean±SD	35.7±3.4
Systolic blood pressure, mm Hg, mean±SD	115±14.5
Diastolic blood pressure, mm Hg, mean±SD	75.8±8.4
Disease course, month, mean±SD	41±11
Erythrocyte sedimentation rate, mm/h, mean±SD	27.3±25.7
C‐reactive protein, mg/dL, mean±SD	2.2±2.6
Artery involvement by CTPA, N (%)	
Pulmonary artery	
Pulmonary trunk	2 (7.4%)
Left pulmonary artery	6 (22.2%)
Right pulmonary artery	25 (92.6%)
Unilateral pulmonary arteries involved	21 (77.8%)
Systemic artery	
Ascending aorta including arch	5 (18.5%)
Brachiocephalic artery	4 (14.8%)
Carotid artery	5 (18.5%)
Subclavian artery	7 (25.9%)
Thoracic aorta	1 (0.04%)
Abdominal aorta	3 (11.1%)
Coronary artery	1 (0.04%)
Upper extremity artery	1 (0.04%)

CTPA = computed tomographic pulmonary angiography.

### 3.1 Echocardiographic identification of pulmonary artery involvements

**Figure 1:**
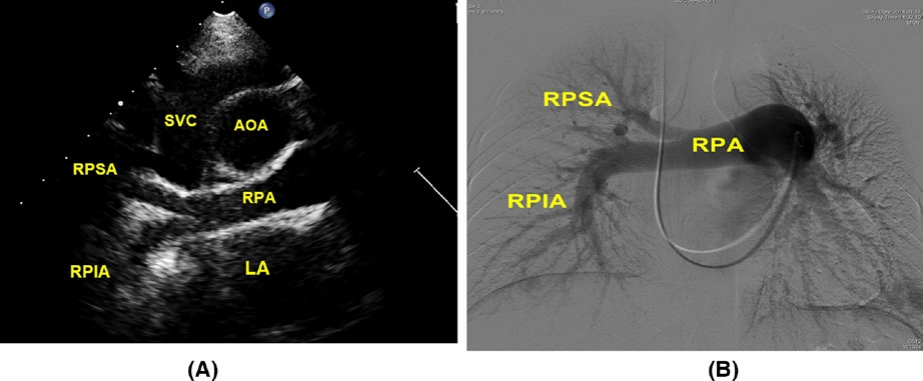
Normal anatomy of the right pulmonary artery with its branches by suprasternal right pulmonary artery long‐axis window (movie clip S1). A. Suprasternal right pulmonary artery long‐axis view showed the right pulmonary artery trunk, RPSA, and RPIA. B. Angiography showed the right pulmonary artery and its branches. SVC = superior vena cava; AOA = aortic arch; RPA = right pulmonary artery; RPSA = right pulmonary superior artery; RPIA = right pulmonary inferior artery; LA = left atrium

In these 27 patients with pulmonary artery involvements shown in CTPA, 25 of them (92.6%) had positive echocardiographic findings. None of echocardiography windows examined in this study could identify pulmonary trunk involvement. Left pulmonary artery involvement was visualized in the parasternal aortic short‐axis view. Right pulmonary artery, middle segment of right pulmonary artery involvement, and right upper lobe artery involvement were better examined in the suprasternal right pulmonary artery long‐axis view (Table 2). An example of the normal anatomy of the right pulmonary artery with its branches by suprasternal right pulmonary artery long‐axis window is shown in Figure 1.

**Table 2:** Identification of pulmonary artery involvements by echocardiography

Pulmonary artery involvements identified by CTPA (N)	Pulmonary artery involvements identified by echocardiography (N, %)		
	Suprasternal right pulmonary artery long‐axis view	Parasternal aortic short‐axis view	Subxiphoid view
Pulmonary trunk (2)	0, 0%	0, 0%	0, 0%
Left pulmonary artery (6)	0, 0%	4, 66.7%	0, 0%
Right pulmonary artery (25)	23, 92%	18, 72%	12, 48%
Middle segment of right pulmonary artery (14)	6, 42.3%	0, 0%	0, 0%
Right upper lobe artery (18)	5, 27.8%	0, 0%	0, 0%

CTPA = computed tomographic pulmonary angiography.

### 3.2 Echocardiography characteristics

**Figure 2:**
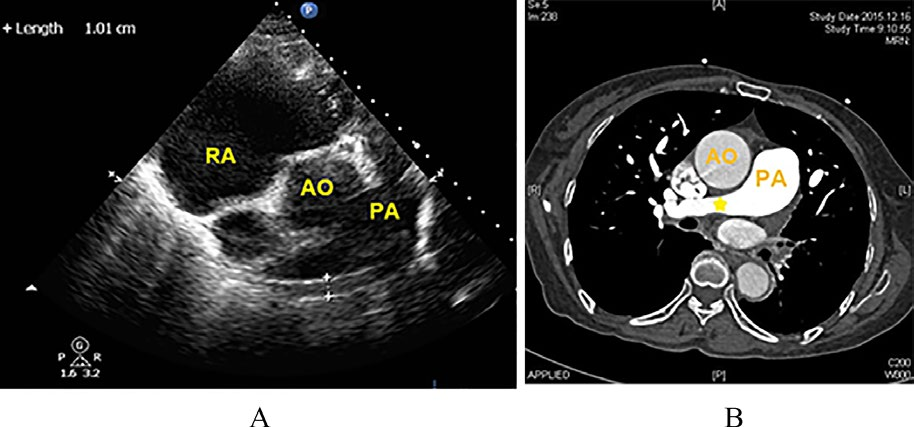
A. Subxiphoid view showed intimal thickening (1cm) in right pulmonary artery and luminal gradual narrowing (movie clip S2). B. CTPA showed right pulmonary artery narrowing (asterisk *). RA = right atrium; AO = aorta; PA = pulmonary artery; CTPA = computed tomographic pulmonary angiography

**Figure 3:**
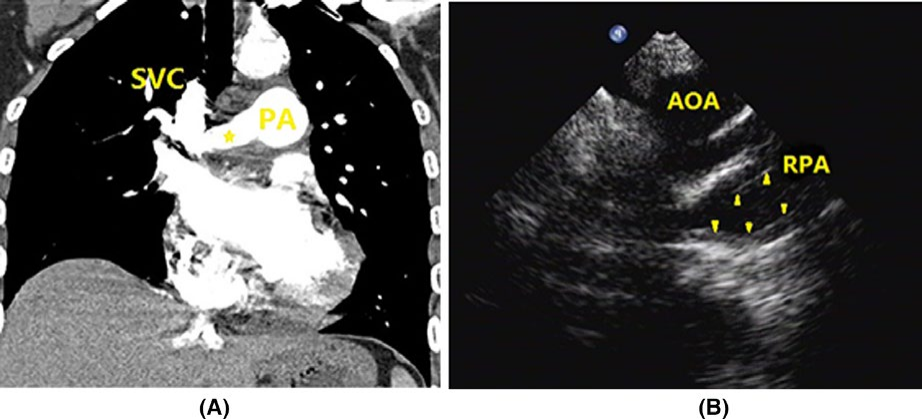
A. Coronal view by CTPA showed right pulmonary artery narrowing (asterisk *). B. Suprasternal right pulmonary artery long‐axis view showed right pulmonary artery extensive intimal thickening and luminal stenosis (arrowheads). SVC = superior vena cava; RPA = right pulmonary artery; AOA = aortic arch; CTPA = computed tomographic pulmonary angiography

**Figure 4:**
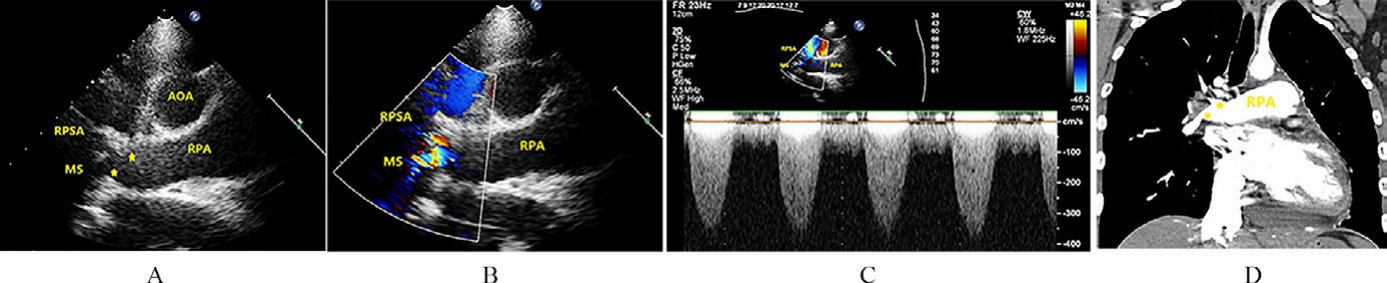
A. Suprasternal right pulmonary artery long‐axis view showed right pulmonary artery intimal thickening in RPSA and middle segment of right pulmonary artery (movie clip S3). B. CDFI showed multicolored mosaic flow signals in RPSA and middle segment of right pulmonary artery. C. CW showed high velocity of blood flow in the middle segment of right pulmonary artery. D. Coronal view by CTPA showed RPSA and RPIA narrowing (asterisk *). AOA = aortic arch; RPA = right pulmonary artery; RPSA = right pulmonary superior artery; MS of RPA = medial segment of the right pulmonary artery; RPIA = right pulmonary inferior artery; CDFI = color Doppler flow imaging; CW = continuous wave; CTPA = computed tomographic pulmonary angiography

**Figure 5:**
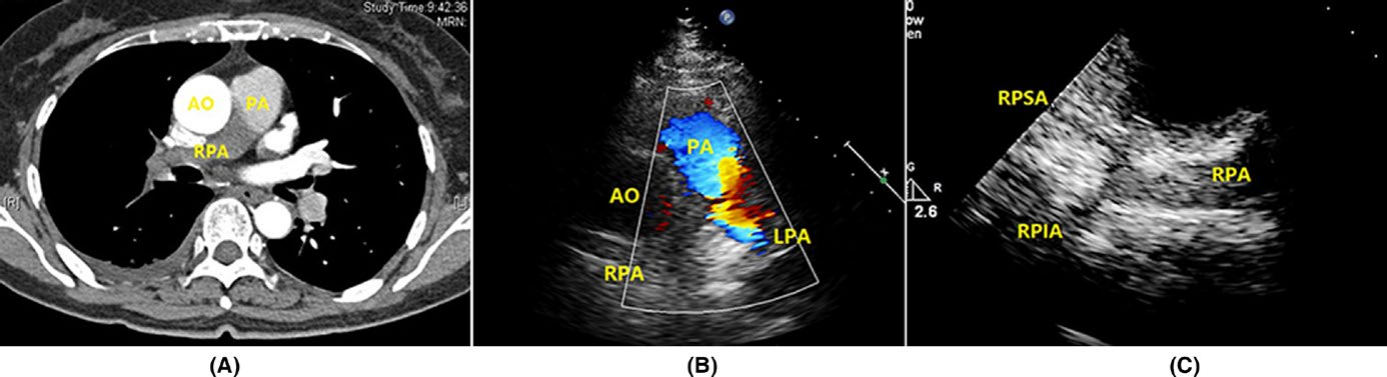
A. CTPA showed filling defects and luminal occlusion in right pulmonary artery. B. Parasternal aortic short‐axis view showed right pulmonary artery filling defects by CDFI. C. Suprasternal right pulmonary artery long‐axis view showed luminal medium echogenic signals and luminal occlusion in right pulmonary artery (movie clips S4 and S5). AO = aorta; PA = pulmonary artery; RPSA = right pulmonary superior artery; RPIA = right pulmonary inferior artery; LPA = left pulmonary artery; RPA = right pulmonary artery; CDFI = color Doppler flow imaging; CTPA = computed tomographic pulmonary angiography

**Figure 6:**
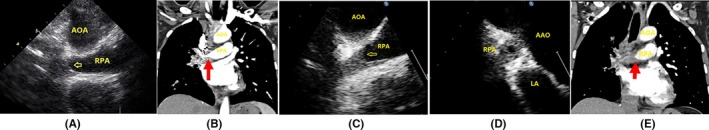
A. The suprasternal right pulmonary artery long‐axis view showed gradually occlusion of distal part of right pulmonary artery (arrowheads) (movie clip S6). B. Coronal view by CTPA showed “rat‐tail” stenosis in distal part of right pulmonary artery but no obvious filling defects in its branches (arrowheads). C. After treatment for 4 months, the suprasternal right pulmonary artery long‐axis view showed worsening distal occlusion in right pulmonary artery which extended into the proximal part (arrowheads) (movie clip S7). D. Suprasternal right pulmonary artery short‐axis view showed annular wall thickening in right pulmonary artery. E. At the same time, repeated CTPA showed luminal occlusion extended into proximal part in right pulmonary artery (arrowheads). AOA = aortic arch; RPA = right pulmonary artery; SVC = superior vein cava; LA = left atrium; CTPA = computed tomographic pulmonary angiography

**Figure 7:**
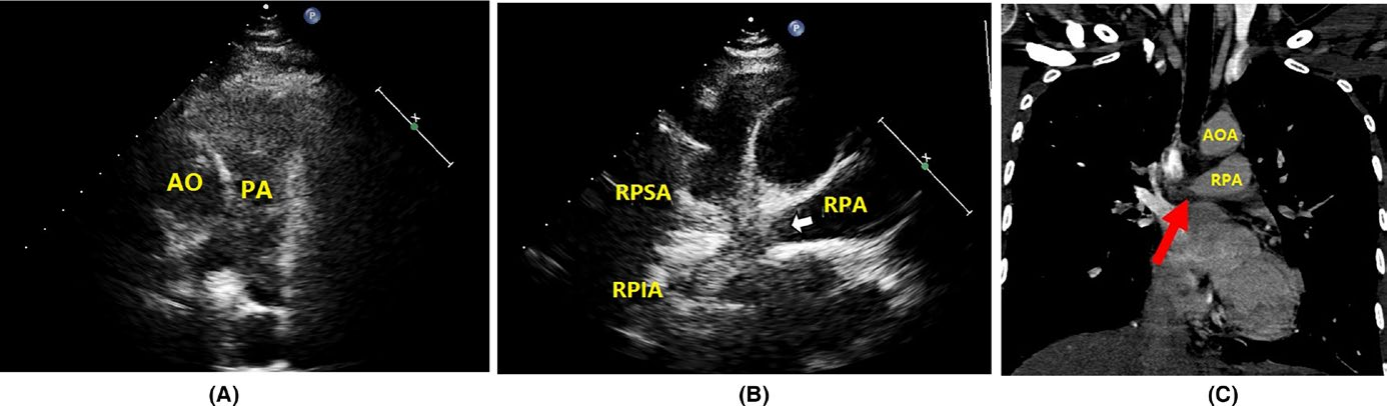
A. Parasternal view showed no obvious abnormalities in right pulmonary artery. B. Suprasternal right pulmonary artery long‐axis view showed high echogenic signals and luminal occlusion in distal right pulmonary artery and RPSA, RPIA (arrowheads) (movie clip S8). C. Coronal view by CTPA showed rat‐tail like stenosis and occlusion in right pulmonary artery (arrowheads). AO = aorta; AOA = aortic arch; PA = pulmonary artery; RPSA = right pulmonary superior artery; RPIA = right pulmonary inferior artery; CTPA = computed tomographic pulmonary angiography

**Figure 8:**
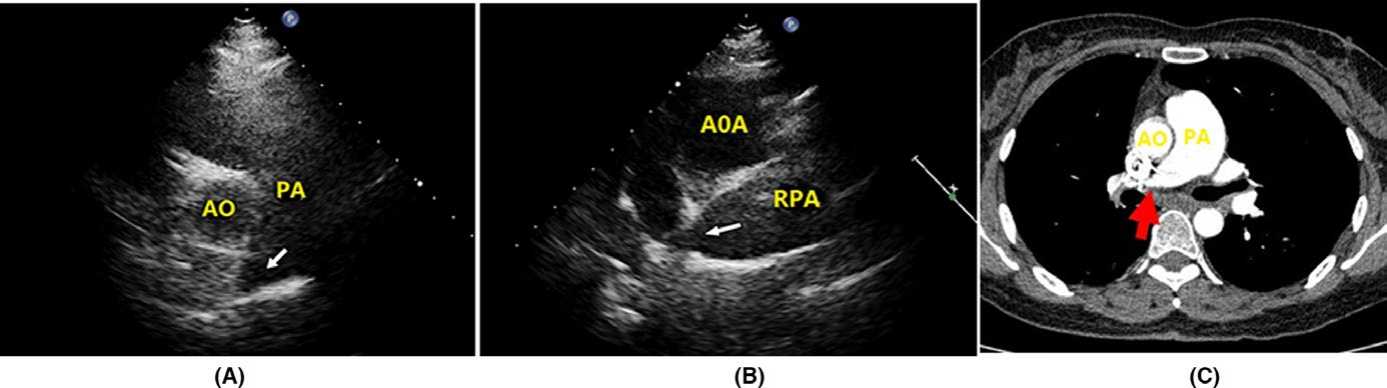
A. Parasternal aortic short‐axis view shows the short right pulmonary artery which gradually narrowed into a blind end (arrowheads). B. Suprasternal right pulmonary artery long‐axis view showed widening of proximal part of right pulmonary artery, and gradual narrowing in its distal part (arrowheads). C. CTPA showed “rat‐tail” like narrowing in right pulmonary artery and occlusion in RPMA, RPIA (arrowheads). AO = aorta; AOA = aortic arch; PA = pulmonary artery; CTPA = computed tomographic pulmonary angiography; RPMA = right pulmonary medial artery; RPIA = right pulmonary inferior artery

**Figure 9:**
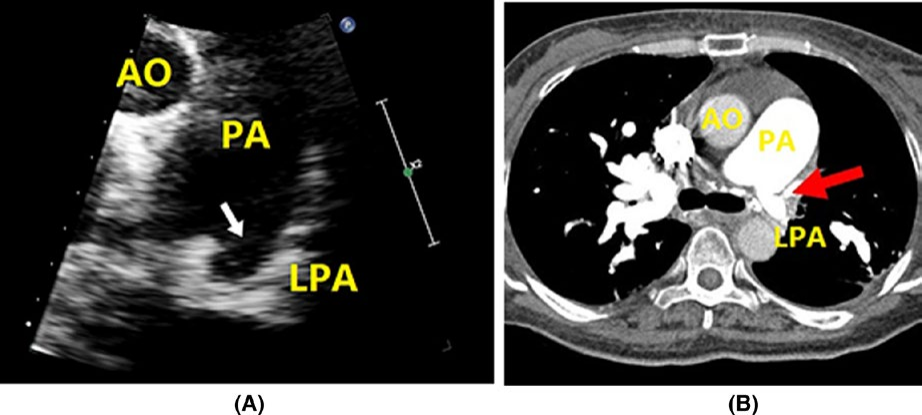
A. Parasternal aortic short‐axis view showed short left pulmonary artery and blind end (arrowheads) (movie clip S9). B. CTPA showed short left pulmonary artery and occluded left pulmonary inferior artery (arrow heads). AO = aorta; PA = pulmonary artery; LPA = left pulmonary artery

Echocardiography revealed specific changes in pulmonary arteries in 25 patients. Luminal stenosis and occlusion were observed in all 25 patients, in which seven patients had intimal thickening and luminal stenosis, 15 patients had luminal occlusion with luminal medium‐to‐high echogenic signals, and 18 patients had gradual narrowing of the distal vessel to an end or short lumen (Table 3). Examples of luminal stenosis and intimal thickening are demonstrated in Figures 2, 3 and 4. Examples of luminal occlusion with luminal medium‐to‐high echogenic signals are demonstrated in Figures 5, 6 and 7. Examples of the gradual narrowing of the distal vessel to an end or short lumen are shown in Figures 8 and 9.

**Table 3:** Identification of characteristic changes by echocardiography in pulmonary artery involvement

Echocardiographic characteristics of pulmonary artery	Number of changes identified by echocardiography (N)	
	Suprasternal right pulmonary artery long‐axis view	Parasternal aortic short‐axis view
Left pulmonary artery		
I Intimal thickening, luminal stenosis	0	0
II Luminal occlusion with luminal medium‐to‐high echogenic signals	0	1
III Gradual narrowing of the distal vessel to an end or short lumen	0	3
Right pulmonary artery		
I Intimal thickening, luminal stenosis	6	1
II Luminal occlusion with luminal medium‐to‐high echogenic signals	14	6
III Gradual narrowing of the distal vessel to an end or short lumen	17	11

## 4 Discussion

Up to 50% patients with Takayasu arteritis could have pulmonary artery involvement. An effective and noninvasive diagnostic method is required to evaluate pulmonary arteries. Previous studies have only reported transthoracic ultrasound evaluations for carotid, brachiocephalic, and subclavian vascular changes. To date, no studies have investigated pulmonary artery involvement via ultrasound. In the present study, we evaluated the echocardiographic characteristics of pulmonary arteries in patients with Takayasu arteritis. Our results suggested that echocardiography could be the method to examine pulmonary artery involvement. Clinicians should pay more attention to the pulmonary artery and its branches during echocardiographic examination in order to facilitate early diagnosis to these patients.

Among the 27 patients identified with Takayasu arteritis and pulmonary artery involvement by CTPA, 92.6% (25 patients) had right pulmonary artery lesions and only 7.4% (two patients) had pulmonary trunk involvement. This was consistent with previous reports that revealed that the right pulmonary artery was more likely to be involved than the left pulmonary artery and that pulmonary trunk involvement was rare by nonechocardiographic methods.^3^, ^4^

In patients with Takayasu arteries, the pathological changes of pulmonary arteries were similar to systemic artery involvements, which include granulomatous panarteritis with adventitial thickening, cellular infiltration of the tunica media, intimal hyperplasia from myofibroblast proliferation, and lumen stenosis and occlusion from fibrosis of the tunica media and intima.^16^, ^17^ The ultrasound characteristic of arteritis was the “macaroni sign,” which was described as diffuse homogeneous echoic circumferential vessel wall thickening.^18^ There were three main echocardiographic features in pulmonary artery involvement. The first type was artery intimal thickening and luminal stenosis (Figures 2, 3 and 4). The typical lesion identified by echocardiography is a long, smooth, homogeneous thickening of the pulmonary arterial wall. The second type was luminal occlusion with luminal medium‐to‐high echogenic signals (Figures 5, 6 and 7). Histopathological examination could reveal the replacement of normal vessel lumen by loose fibrosis tissue and is presented as “vessel‐in‐vessel” pathologic.^3^ Patients with this type of lesion have increased risk for site thrombosis. The third type was the gradual narrowing of the distal vessel to an occluded end or short residue lumen (Figures 8 and 9). This typical vessel lesion was described as “rat‐tail stenosis” in pulmonary angiography and CTPA studies, which indicates significant distal vessel stenosis or occlusion.

Previous studies reported that luminal occlusion was the most common finding in pulmonary artery involvement examined by CTPA.^3^ In the present study, we more frequently observed luminal medium‐to‐high echogenic signals. This might be due to the fact that CT has relatively poor resolution to small vessels, whereas echocardiography has higher resolution compared to CTPA (10 times than CTPA) and is more sensitive to detect soft tissue and luminal changes.^9^ This might imply that echocardiography could reveal a more detailed arterial wall and luminal lesion compared to computed tomographic study. In addition, in patients with luminal lesions and artery stenosis or occlusion, CTPA can show its filling defect by contrast agent, but has difficulty in providing direct evidence of intimal thickening and distinguishing the thickened intima from the extravascular soft tissue (Figures 2, 3, 4, 5, 6, 7). This filling defect could be mistaken for other diseases such as pulmonary embolism or pulmonary tumor.^19^ Echocardiography could clearly identify lesions in lumen and three layers of vessel (intima, media, adventitia) and distinguish them from adjacent soft tissues and luminal thrombosis. It might provide evidence for early pulmonary artery involvement in patients with Takayasu arteritis and help in the diagnosis and differential diagnosis of Takayasu arteritis.

Despite its high resolutions, echocardiography examination also has its limitations. The biggest limitation is its low penetration through bone or gas. We were not able to identify pulmonary trunk lesions. This might be due to the location of the pulmonary trunk (behind the sternum) and influences from the air in the lung. In addition, the direction of the pulmonary trunk is parallel with the echocardiographic beam in the parasternal aortic short‐axis window, which makes it difficult to reveal lesions to the artery. Left pulmonary artery involvement was only identified through the parasternal aortic short‐axis window. In this window, only the proximal part of the left pulmonary artery was examined. The distal part of the artery was not able to be visualized due to its deep anatomy location. The right pulmonary artery, as well as its branch involvement, was best observed in the suprasternal right pulmonary artery long‐axis view. In this view, the transducer was close to the pulmonary artery trunk and the beams were perpendicular to the right pulmonary artery. Both right pulmonary artery and its branches could be visualized very well in this window. The initial segment of right pulmonary artery lesions could also be revealed in the parasternal aortic short‐axis view and subxiphoid view. In patients with pulmonary disease, such as emphysema, subxiphoid view could visualize right pulmonary artery lesions.

The limitations of the present study were as follows: the retrospective nature of the study, single central study, and the small number of patients. We only selected patients with abnormal CTPA findings in the pulmonary artery. Therefore, we were not able to report false positive results of echocardiography. Individual performance variations by sonographers could also affect our results. Future studies with large sample size are required to confirm our findings in the current study.

## 5 Conclusions

Echocardiography is a noninvasive test and could provide evidence of characteristic changes in intimal thickening, luminal lesions, and luminal stenosis in pulmonary arteries in patients with Takayasu arteritis. Left pulmonary artery involvement was revealed in the parasternal aortic short‐axis view. Right pulmonary artery involvement was best observed in the suprasternal right pulmonary artery long‐axis view, with complementary views from the parasternal aortic short‐axis and subxiphoid angles.

## Supporting information


**Movie clip S1.** Normal anatomy of the right pulmonary artery with its branches by suprasternal right pulmonary artery long‐axis window.

**Movie clip S2.** Subxiphoid view showed intimal thickening in right pulmonary artery and luminal gradual narrowing.

**Movie clip S3.** Suprasternal right pulmonary artery long‐axis view showed right pulmonary artery intimal thickening in RPSA and middle segment of right pulmonary artery.

**Movie clip S4.** Suprasternal right pulmonary artery long‐axis view showed luminal medium echogenic signals and luminal occlusion in right pulmonary artery.

**Movie clip S5.** Suprasternal right pulmonary artery short‐axis view showed luminal occlusion in right pulmonary artery by CDFI.

**Movie clip S6.** The suprasternal right pulmonary artery long‐axis view showed gradually occlusion of distal part of right pulmonary artery.

**Movie clip S7.** After treatment for 4 months, the suprasternal right pulmonary artery long‐axis view showed worsening distal occlusion in right pulmonary artery which extended into the proximal part.

**Movie clip S8.** Suprasternal right pulmonary artery long‐axis view showed high echogenic signals and luminal occlusion in distal right pulmonary artery and RPSA, RPIA.

**Movie clip S9.** Parasternal aortic short‐axis view showed short left pulmonary artery and blind end.
